# Communicating cancer treatment with pictogram-based timeline visualizations

**DOI:** 10.1093/jamia/ocae319

**Published:** 2025-01-16

**Authors:** Helena Klara Jambor, Julian Ketges, Anna Lea Otto, Malte von Bonin, Karolin Trautmann-Grill, Raphael Teipel, Jan Moritz Middeke, Maria Uhlig, Martin Eichler, Sebastian Pannasch, Martin Bornhäuser

**Affiliations:** National Center for Tumor Diseases, University Cancer Center, NCT-UCC, Universitätsklinikum Carl Gustav Carus an der Technischen Universität Dresden, 01307 Dresden, Germany; Institute for Data Analysis, Visualisation and Simulation, DAViS, University of Applied Sciences of the Grisons, Chur 7000, Switzerland; National Center for Tumor Diseases, University Cancer Center, NCT-UCC, Universitätsklinikum Carl Gustav Carus an der Technischen Universität Dresden, 01307 Dresden, Germany; Engineering Psychology and Applied Cognitive Research, Faculty of Psychology, Technische Universität Dresden, 01069 Dresden, Germany; Engineering Psychology and Applied Cognitive Research, Faculty of Psychology, Technische Universität Dresden, 01069 Dresden, Germany; Medical Clinic 1, Universitätsklinikum Carl Gustav Carus an der Technischen Universität Dresden, 01307 Dresden, Germany; Medical Clinic 1, Universitätsklinikum Carl Gustav Carus an der Technischen Universität Dresden, 01307 Dresden, Germany; Medical Clinic 1, Universitätsklinikum Carl Gustav Carus an der Technischen Universität Dresden, 01307 Dresden, Germany; Medical Clinic 1, Universitätsklinikum Carl Gustav Carus an der Technischen Universität Dresden, 01307 Dresden, Germany; Medical Clinic 1, Universitätsklinikum Carl Gustav Carus an der Technischen Universität Dresden, 01307 Dresden, Germany; National Center for Tumor Diseases, University Cancer Center, NCT-UCC, Universitätsklinikum Carl Gustav Carus an der Technischen Universität Dresden, 01307 Dresden, Germany; Engineering Psychology and Applied Cognitive Research, Faculty of Psychology, Technische Universität Dresden, 01069 Dresden, Germany; National Center for Tumor Diseases, University Cancer Center, NCT-UCC, Universitätsklinikum Carl Gustav Carus an der Technischen Universität Dresden, 01307 Dresden, Germany; Medical Clinic 1, Universitätsklinikum Carl Gustav Carus an der Technischen Universität Dresden, 01307 Dresden, Germany

**Keywords:** oncology, treatment communication, visual aids, data visualization, health communication

## Abstract

**Objective:**

This study evaluated the legibility, comprehension, and clinical usability of visual timelines for communicating cancer treatment paths. We examined how these visual aids enhance participants’ and patients’ understanding of their treatment plans.

**Materials and Methods:**

The study included 2 online surveys and 1 in-person survey with hematology cancer patients. The online surveys involved 306 and 160 participants, respectively, while the clinical evaluation included 30 patients (11 re-surveyed) and 24 medical doctors. Participants were assessed on their ability to understand treatment paths provided with audio information alone or with visual aids. The study also evaluated the comprehensibility of key treatment terms and the ability of patients to recall their cancer treatment paths.

**Results:**

Visual representations effectively communicated treatment terms, with 7 out of 8 terms achieving over 85% transparency as pictograms, compared to 5 out of 8 for comics and 4 out of 8 for photos. Visual treatment timelines improved the proportion of correct responses, increased confidence, and were rated higher in information quality than audio-only information. In the clinical evaluation, patients showed good comprehension (mean proportion correct: 0.82) and recall (mean proportion correct: 0.71 after several weeks), and both patients and physicians found the visual aids helpful.

**Discussion:**

We discuss that visual timelines enhance patient comprehension and confidence in cancer communication. We also discuss limitations of the online surveys and clinical evaluation. The importance of accessible visual aids in patient consultations is emphasized, with potential benefits for diverse patient populations.

**Conclusion:**

Visual aids in the form of treatment timelines improve the legibility and comprehension of cancer treatment paths. Both patients and physicians support integrating these tools into cancer treatment communication.

## Introduction

The National Academy of Medicine/United States defines high-quality care as encompassing safety, effectiveness, timeliness, efficiency, patient-centeredness, and equity.^1^ Important for patient-centeredness and equity is an effective communication between health care providers and patients.^2–4^ Comprehensible information and patients’ health literacy, ie, the ability to understand written and verbal medical information about diagnosis, prognosis, uncertainties, and risks, are important in shared decision-making.^5^ However, mismatches in numeracy, literacy, and experience frequently challenge physicians’ communication with patients.^6^ Around 10% of the global population is estimated to lack basic literacy and, at a lower percentage, also numeracy skills, and even among those with high school education, adults have comprehension difficulties.^7–9^ Additionally, medical teams often encounter non-native speakers and patients with cognitive decline due to age or neurotoxic therapies, raising concerns about their understanding of treatment regimens for informed decision-making and further challenging the process.^5^

Health literacy gaps are well-documented obstacles to equitability in care. Consent forms are frequently written in inaccessible language and illegible print.^3^^,^^10^ Likewise, verbal communication is often overly complex, with medical teams often overestimating patients’ literacy levels.^11–14^ This complexity is exacerbated when discussing intricate medical information, such as cancer treatments.^15–17^ Consequently, studies consistently find that patients tend to recall only half of their medical information,^17–22^ leading to implications for patients safety, treatment adherence and health outcomes.^12^^,^^23^^,^^24^

Visual aids have proven to be beneficial for understanding the data, especially in the case of risks, uncertainties, and numerical information.^25–27^ In health care, visual aids are beneficial when promoting healthy choices to improving treatment adherence and risk-avoidance.^28–32^ Information that is supplemented by comics or pictograms measurably enhances health understanding and is perceived as helpful by patients.^30^^,^^33^^,^^34^ This approach is particularly helpful for vulnerable and non-native speaking patients, with whom visual aids are more effective even than translations.^30^^,^^33^ Despite these advantages, visual aids are at present underutilized in patient communication.^35^^,^^36^ The overall aim of this study was to develop and evaluate visual timelines for communicating cancer treatment paths using 3 hematological neoplasms as case studies.

## Methods

**Figure 1.**
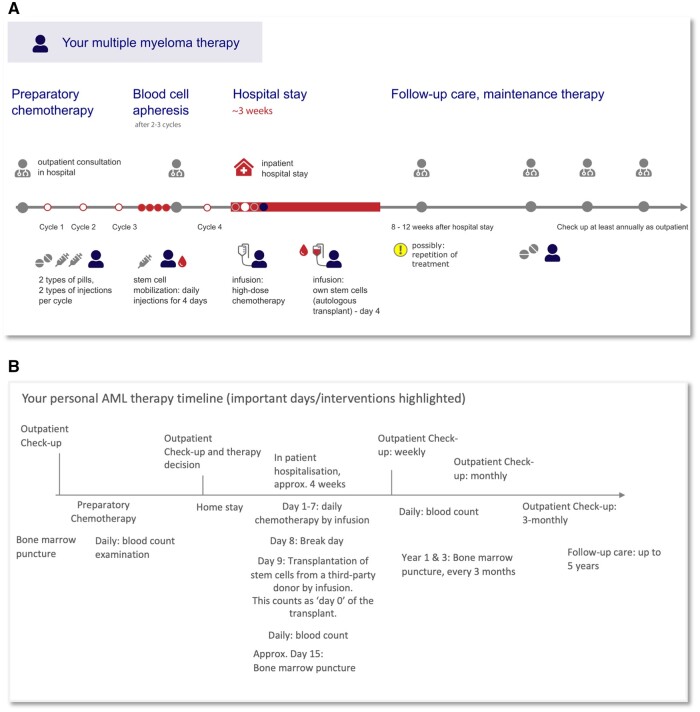
(A) Example of a pictogram-based visual treatment timeline co-designed with patients and evaluated for comprehension with participants and patients. Multiple myeloma treatment with high-dose chemotherapy and autologous stem cell transplantation. (B) Example of text-based visual timeline (Study 2).

To develop visual aids in the form of visual treatment paths for patients with hematological neoplasm (Figure 1A, Figure S3), we assessed the information needs by observing outpatient consultations as well as via meetings with patients, patient board, and clinicians, and guides for patient treatment plans and schedules.^37^ This revealed the time-course of treatment, the sequence of interventions, and their settings (hospital stay or outpatient care) as relevant areas for visual aids. In existing public information from national cancer institutes and cancer charities in the United States, United Kingdom, and Germany treatment timeline visualizations^38^ were scarce in the text-heavy brochures, and mostly limited to photos and anatomical illustrations (Table S1). Only 3 of the 44 figures provided some information on the treatment timelines.

Using an iterative design approach^39–42^ that included the intended audiences,^6^ we then developed visual treatment timelines, the final version of which was used in this study. In this work, we evaluate the usability of the visual treatment timelines. In the first step, Study 1 compared the legibility of different visual representations for key terms. Next, Study 2 was used to assess participants’ comprehension when receiving information either through audio alone or supplemented with text- or pictogram-based visual treatment timelines. Studies 1 and 2 were conducted with participants in an anonymous online survey and not with patients. Finally, Study 3 evaluated the clinical use of visual treatment timelines with patients. Overview of study designs: Figure S1.

### Study 1: design and evaluation of visual representations

#### Design

Essential for designing a visual aid is the identification of suitable visual representations to encode the key terms. We have selected pictograms for the visual treatment timelines as they are widely used eg, in public transport,^41^ have been integrated in health information,^31^^,^^43^^,^^44^ and are highly rated by patients.^34^^,^^45^^,^^46^ However, given their high abstraction level, pictograms must be evaluated before use with the intended audience.^47^ By ANSI (American National Standards Institute) requirements, only visuals that are recognizable by at least 85% of participants fulfil the criteria for being self-explanatory and helpful.^47^ Alternative visual representations are photographs and comics, but these may contain irrelevant information, eg, gender of medical professional, or overemphasize details (comics).

To compare visual representations, we designed a one-factorial (2 phases) within-subjects design. In each phase, 8 terms (Figure S2) were shown in 3 different visual representations (pictogram, comic, photo). To minimize the order effect, the visual representations were shown in random order. The participants were required to answer questions on the transparency (phase 1) and translucency (phase 2) of the visual representations. To assess transparency (guessability, Question: What is the meaning of the prompted visual?), participants were required to enter free text to describe a visual representation with 1 term. This was matched to a corpus/syntax of correct terms. To examine translucency (Question: “Is the prompted visual suitable for term?”), participants rated the suitability of the visual representation and its term on a scale from 1 to 7 (1-4: not appropriate; 5-7: appropriate). The participants’ health literacy was tested with 3 control questions to monitor a possible selection bias (validated test from^48^). The study was preregistered at OSF (https://osf.io/cs57n).

#### Participants and power analysis, procedure

Inclusion criteria were ability to understand, read, and write German. No personal data was collected so that the identity of the participants was completely protected. Since no identifiable personal information was obtained, this survey did not fall under the requirements for ethical review board approval at the TU Dresden. We targeted a sample size of 259 based on a power analysis with a desired power of 0.8, alpha level of 0.05, and an assumed medium effect size of 0.1. Based on inclusion/exclusion criteria, we included 306 participants (mean age 39) in the study, see Table 1 for cohort description.

**Table 1.** Cohort descriptions.

Feature	Category	Number	Percent (%)
**Study 1** Transparency and translucency of visual representations			
Age	<20	13	4
	20-39	163	53
	40-59	99	32
	60+	31	10
Gender	Male	72	24
	Female	228	75
	Diverse	3	1
	Not answered	3	1
Health literacy^48^	High	290	95
	Low	16	5
**Study 2** Information delivery formats for cancer treatment timelines			
Groups	Audio (a)	60	38
	Pictogram (p)	47	29
	Text (t)	53	33
Age	<18	1 (p: 1, t: 0, a: 0)	<1
	18-30	66 (p: 18, t: 26, a: 22)	41
	31-60	85 (p: 25, t: 27, a: 33)	53
	60+	7 (p: 2, t: 1, a: 4)	4
	Not answered	1 (p: 1, t: 0, a: 0)	<1
Cancer knowledge	Large	19 (p: 5, t: 7, a: 7)	12
	Some	82 (p: 23, t: 34, a: 25)	51
	None	58 (p: 19, t: 12, a: 27)	36
	Not answered	1 (p: 0, t: 1, a: 0)	<1
**Study 3** Clinical evaluation of visual treatment timelines			
Gender	Male	19	63
	Female	11	36
Age	40-49	5	17
	50-59	12	40
	60-69	11	37
	> 70	2	7
Disease entity	Multiple myeloma	21	70
	Lymphoma	6	20
	AML	3	10

The online, open-label cohort questionnaires were conducted in German, administered using LimeSurvey software, and piloted to validate questions and solve technical issues. Online participants were recruited via social media, notice boards, and university mailing-lists. Participants had to provide informed consent, agree to anonymous responses being used for research, were provided with contact information of researchers, and the opportunity to withdraw.

#### Statistical analysis and data visualization

Data on transparency and translucency were analyzed using SPSS Version 28.0.0.0. For the transparency we assessed the frequency of correct answers. For the translucency participants’ ratings were assigned a numerical value (not appropriate: 0, appropriate: 1) and then summarized by frequency for each term.

### Study 2: comparison of information delivery formats for cancer treatment timelines

#### Design

We compare the effectiveness of 3 formats for delivering information on cancer treatment paths with an between-subject design with multiple comparisons. Participants were randomly assigned to 1 of the 3 groups, each corresponding to a different treatment condition (the primary independent variable): A, audio only (scenario in current patient consultation without reading materials), P, audio with pictogram-based treatment timeline, or T, audio with text-based treatment timeline (Figure 1A and B, Figure S3). Group P and T received time course data as flow-chart/timeline and they were tasked to identify specific information with access to the stimulus and to rate their trust/confidence.^49^ All participants received some general orienting information about leukemia before starting the survey and a 2-minute audio information about treatment and timelines. Participants then answered 10 content questions (multiple choice, see Table S3). After each content question, participants were asked to rate their confidence in answering (4-step Linkert rating scale, “How confident are you in your answer?,” answers: unsure, somewhat unsure, somewhat sure, sure). Participants with pictogram/text-based treatment timelines could use the respective visual aids while answering questions.

After the completion, participants were asked to rate the quality of the received information using a 4-step Linkert scale (answers: incomprehensible, rather incomprehensible, rather understandable, understandable). We included a question on prior health education to monitor a potential selection bias (self-assessment of prior knowledge on cancer, answers: no/some/extensive prior knowledge). The study was preregistered at OSF (https://osf.io/t2gkq). The timing of each step was recorded.

#### Participants and power analysis, procedure

The inclusion criteria were identical to that of Study 1. To obtain reliable differences in responses, we set the desired statistical power at 0.8 and chose an alpha level of 0.05 and, given the lack of previous studies, assumed a medium effect size of 0.25, which revealed a required sample size of 159. 160 participants were included in the analysis of the effect of visual treatment timelines (mean age 38, Table 1). The same software and the same procedure were used as in Study 1.

#### Statistical analysis and data visualization

Data were analyzed using SPSS Version 28.0.0.0. The effectiveness of the information delivery formats was evaluated by calculating several key parameters.

To assess individual-level comprehension, specifically, identification of key information,^49^ we calculated the proportion of correct responses for each participant. To assess the difficulty of each of the 10 questions, we calculated the proportion of participants who answered correctly. To assess the total response times (content question, confidence/control questions) the time spent on survey questions was summed up. To analyze confidence rating, Linkert scale responses were assigned a corresponding numerical value (1—unsure, 2—somewhat unsure, 3—somewhat sure, 4—sure), and averaged per participant across all answers and normalized to a continuous 0 (low) to 1 (highest) scale to be comparable in scale to the other observations. To compare the rating of information quality, the Linkert scale responses were assigned a corresponding numerical value (1—incomprehensible, 2—rather incomprehensible, 3—rather understandable, 4—understandable) and then transformed to a 0 (lowest) to 1 (highest) scale to be comparable in scale to the other observations (0—lowest rating, 0.33—slightly positive rating, 0.67—moderately positive rating, 1—highest rating).

A one-way ANOVA was employed to compare the means of the 3 groups to test for statistically significant group effects, and variance homogeneity was confirmed with Levene’s test. If Levene’s test indicated a difference in variances, we then performed a Tukey post hoc test to determine which specific group differences were statistically significant.

### Study 3: clinical evaluation of visual treatment timelines

#### Design

We evaluated the clinical usability of visual treatment timelines for cancer treatment timeline communication with a non-blinded, open-label patient questionnaire. The questionnaire included 5 multiple-choice content questions (a subset of the questions similar to those used in Study 2, see Table S3), 3 questions with rating scales (5-step Likert scale, usefulness of the visual aid, answers: very/somewhat/not helpful, distracting, not sure), and 1 open-ended question (“How do you envision to/did you use the visual treatment timelines at home?”). The questionnaire was piloted and validated with medical doctors (MDs) and patient members of the patient board of the National Center for Tumor Diseases/NCT-Dresden. The clinical evaluation, including the patients consent form, was approved by the TU Dresden ethics board (BO-EK-338072022). Patients received time course data as flow-chart/timeline and were tasked to recall and rate the information.^49^

#### Participants

All patients presenting with hematological neoplasms were invited to participate in the study. Inclusion criteria: confirmed diagnosis of a hematologic neoplasm, age ≥18 years at diagnosis, attended at the Medical Clinic 1, University Hospital Dresden, ability to provide informed consent. Exclusion criteria: inability to complete a structured questionnaire, eg, comorbid dementia, insufficient language proficiency, illiteracy. Patients were informed on the purpose and design of the study (informed consent in compliance with the TU Dresden ethics board). No statistical tests were planned for this clinical evaluation, thus power analysis was not required.

In total 34 patients were recruited; 30 met inclusion criteria. Patients treated for multiple myeloma were invited to a re-survey upon returning for a scheduled stem cell apheresis 4-6 weeks after the initial consultation. Before this re-survey, no additional verbal, written, or visual information was provided, and patients were interviewed before their consultation with a medical doctor. 11 patients were included in the re-survey. Survey responses from 24 medical doctors were analyzed (cohort description see Table 1).

#### Procedure

Following informed consent, MDs explained the treatment to patients using the corresponding timeline visualization (Figure 1A, Figure S4). Patients were allowed to keep their timeline visualizations for future reference. Patients then completed the paper-based survey, which included comprehension (identification) questions and ratings of information and could refer to their timeline visualization as needed. MDs also completed a survey after each patient, rating their experience.

#### Statistical analysis and data visualization

For the clinical evaluation, descriptive statistics were used to summarize the frequency of responses.

All data plots were prepared using R and ggplot2, version 4.3.2.^50^^,^^51^

### Patient and public involvement

Our work was supported by the patient board of the National Center for Tumor Diseases/NCT-Dresden, which also includes former patients. The planned work and its progress were presented to the entire board. A project advisory group of 3 board members was also involved in reviewing and piloting the questionnaire and provided helpful input on the design of visual treatment timelines. The ongoing project was presented publicly at “patient day’s” organized by the National Center for Tumor Diseases.

## Results

In consultation with the patient board, the medical team, and based on feedback from our intended audience,^6^ we had developed visual timelines for communicating cancer treatment paths (Figure 1). To evaluate the effectiveness of these visual aids, we: (1) tested the clarity of the pictograms used; (2) assessed patients’ comprehension of treatment paths information with or without these visual timelines; and (3) tested their use in a clinical setting.

### Study 1—transparency and translucency of visual representations

**Figure 2.**
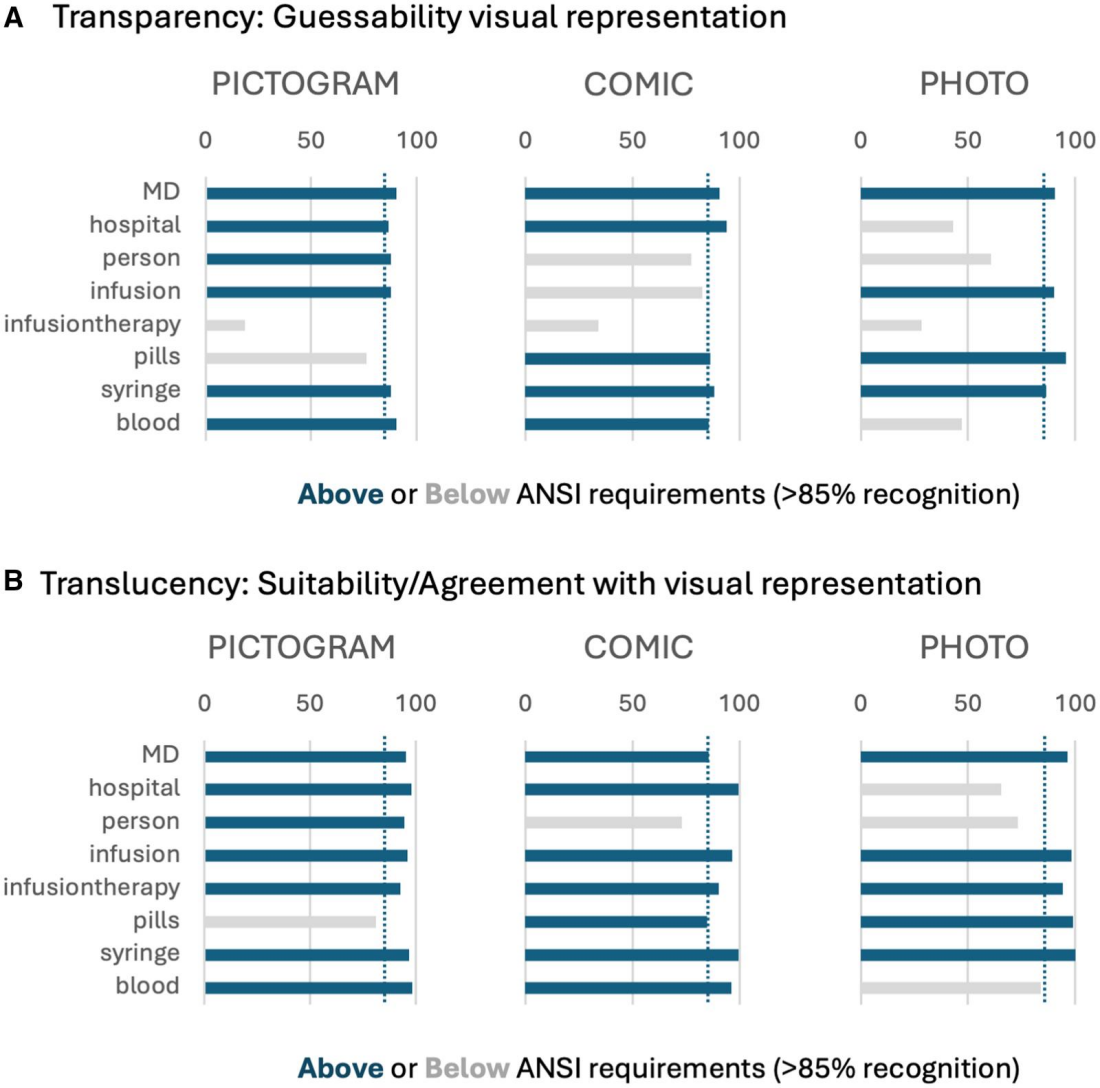
Transparency and translucency of visual representations. (A) % Transparency: can participants guess the image term? Overall, many are above the 85% mark (dashed blue line). Highest number of icons above 85% are pictograms, then comics, lowest photos. (B) % Translucency: do participants rate the icon as suitable for a known term? Overall, many are above the 85% mark. Highest number of icons above 85% are pictograms, then comics, lowest photos. Participants: 306.

We compared pictograms to comics and photos in their effectiveness to communicate medical term (Tables 2 and 3). We tested 8 terms relevant for communicating cancer treatment paths (Figure S2). Of the 8 terms, 6 pictograms, 5 comic representations, and 4 photos were correctly identified (guessed) by at least 85% of the 306 participants and therefore met the American National Standards Institute (ANSI) transparency criterion of being understandable/guessable by at least 85% of participants (Figure 2A). Of the 8 terms, 6 pictograms and comics, and 5 of 8 photos were also rated as suitable by at least 85% of participants, and thus fulfilled the translucency criterion (Figure 2B). Visual representations that did not pass the transparency or translucency criterion were the pictogram for “Pill” (76%), the comic for “Person” (77%), and the photos for “Hospital” (43%), “Person” (60%), and “Blood” (47%), which were neither guessable, nor considered suitable by >85% of participants, the ANSI requirements (85%) for symbols, and most are even below the somewhat more flexible standard of the International Organization for Standardization (ISO) of being understood by at least 67% of users without explanatory text.^47^ The visual representations for “infusion therapy,” arguably a highly specific term, was guessable by only 18% of the participants, but when prompted rated as “very suitable” in all visual representations (90%-94%).

**Table 2.** Summary of transparency and translucency of visual representations (Study 1).

	Transparency (% correct)			Translucency (% correct)		
Term	Pictogram	Photo	Comic	Pictogram	Photo	Comic
MD	**90.2**	**90.6**	**90.6**	**95.4**	**96.4**	**85.7**
Hospital	**86.6**	43.0	**94.1**	**98.0**	65.5	**99.7**
Person	**87.9**	60.6	77.5	**94.5**	73.3	73.0
Infusion	**87.6**	**90.2**	82.7	**96.1**	**98.4**	**96.7**
Infusion therapy	18.6	28.3	33.9	**92.8**	**94.1**	**90.2**
Pills	76.2	**95.8**	**86.3**	81.1	**99.1**	**85.0**
Syringe	**87.6**	**86.3**	**88.3**	**96.7**	**100**	**99.7**
Blood	**90.2**	47.2	**85.7**	**98.4**	84.0	**96.4**

Visual representations that meet the American National Standards Institute (ANSI) requirement (recognizable to at least 85% of participants) are highlighted in bold, those below the threshold are shaded in grey. Included participants: 306.

**Table 3.** Statistical tests transparency of visual representations (Study 1).

Test	Frequencies (*n*)			Cochrane Q test (Q (2), *p*)	Pairwise Chi-square test, significance level (*χ*^2^(2), *p*)		
Term	Picto	Photo	Comic	Across visual representations	Picto: Photo	Picto: Comic	Photo: Comic
MD	277	278	278	0.043, *p* = .979			
Hospital	266	132	289	224.66, *p* < .001	−0.436, *p* ≤ .001	0.075, *p* = .126	0.511, *p* ≤ .001
Person	270	186	238	83.60, *p* < .001	0.274, *p* ≤ .001	0.104, *p* < .05	−0.169, *p* ≤ .001
Infusion	269	277	254	14.35, *p* < .001	−0.026, *p* = .583	0.049, *p* < .05	0.075, *p* ≤ .001
Infusion-therapy	57	87	104	31.76, *p* < .001	0.098, *p* ≤ .001	0.153, *p* ≤ .001	0.055, *p* = .132
Pills	234	294	265	53.46, *p* < .001	−0.195, *p* ≤ .001	−0.101, *p* ≤ .001	0.094, *p* ≤ .001
Syringe	269	265	271	1.10, *p* = .578			
Blood	277	145	263	195.93, *p* < .01	0.430, *p* ≤ .001	0.046, *p* = .530	−0.384, *p* ≤ .001

Included participants: 306.

This data indicates a slight skew towards pictograms and comics being more guessable and suitable, however based on our 8 tested terms, no visual representation was consistently outperforming the other. “MD” and “Syringe” were equally guessable in all forms of visual representation (Cochrane Q test no deviation across all visual representations, Table 3), while infusion therapy was not sufficiently guessable in any representation. For “Hospital,” “Person,” “Infusion,” and “Blood,” pictograms were significantly more guessable than comics and/or photos as conformed by Chi-squared testing. Only for “Pill,” comic and photo representations significantly outperformed the pictogram. A similar result was obtained for the suitability of visual representations. Again, for “Hospital,” “Person,” and “Blood,” pictograms were rated significantly more suitable than comics and/or photos, however not only “MD” and “Syringe,” but also “Infusion” and “Infusion therapy” were rated equally suitable in all visual representations.

Applying a validated health literacy test^48^ revealed a relatively homogeneous cohort, with over 95% of participants demonstrating high health literacy (cohort description: Table 1). As a result, we could not test differences in visual representation transparency and translucency across literacy levels. Also, as the group sizes across these age categories were not equal, testing for age-dependent could would not reliably reveal effects. We did however observe that elderly participants were generally slower in their responses across all forms of visual representations, and that both younger and older participants responded fastest with pictograms than with photo representations (Table S2).

### Study 2—comparing information delivery formats for cancer treatment timelines

**Figure 3.**
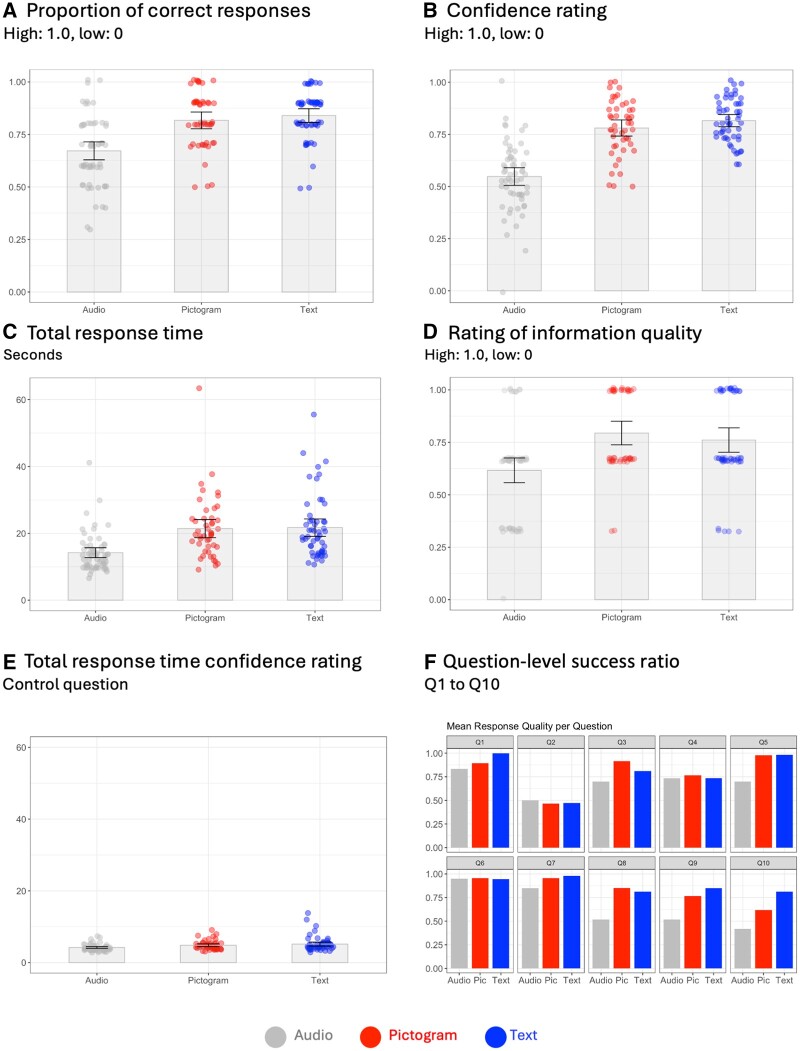
Comparing information delivery formats for cancer treatment paths. Visual aids (pictogram- and text-based) improved proportion of correct responses: (A) the overall proportion of correct responses with a higher value indicating that a question was easier to be answered correctly by participants, (B) increased respondents confidence rating and (C) response times, and (D) were rated higher in information clarity. (E) Response times for content questions varied, while the times for the control questions were similar across groups. (F) Question-level success ratio indicate how easy the individual questions 1-10 (Table S3) were answerable by participants in the 3 groups. Participants: 160 (Group audio only: 60, Group P [pictogram and audio]: 47, Group T [text and audio]: 53).

We compared participants’ ability to understand cancer treatment path information presented as audio only, text treatment timeline, or visual treatment timeline (Figure 1A and B, original German versions: Figure S3, summary of results: Table S4). Compared to participants who received only audio information (*n* = 60), simulating a typical patient consultation, those who also received treatment timelines demonstrated significantly higher proportion of correct responses (suggesting questions were easier to answer) when answering content question (Figure 3A, 0.84/0.82 compared to audio 0.68). This mean accuracy was not statistically different between participants receiving text-based (*n* = 53) and pictogram-based (*n* = 47) treatment timelines (Table 4). Participants with treatment timelines not only were quantitatively better in answering content questions, but also subjectively indicated feeling more confident their answers were correct (Figure 3B, 0.78/0.82 compared to audio 0.55) and also rated the quality of the information higher (Figure 3D, 0.79/0.76 compared to audio 0.62). Overall, the groups with treatment timelines were significantly slower in their response times than participants with audio information only (Figure 3C, Table 4, 21.4/21.7 seconds compare to audio 14.2 seconds). Although the groups were slower in answering content-related questions, in comparison the time they required to rate their confidence in their answers was instead comparable across all 3 groups and not statistically different (Figure 3E, 4.1/4.8/5.1 seconds). Thus, the slower response time may indicate that participants indeed make use of the visual aids when answering questions. Despite this slight delay, the groups with visual treatment timelines, both text and pictogram-based, showed significantly higher overall proportion of correct responses, as well as for a higher question-level success ratio (Figure 3F).

**Figure 4.**
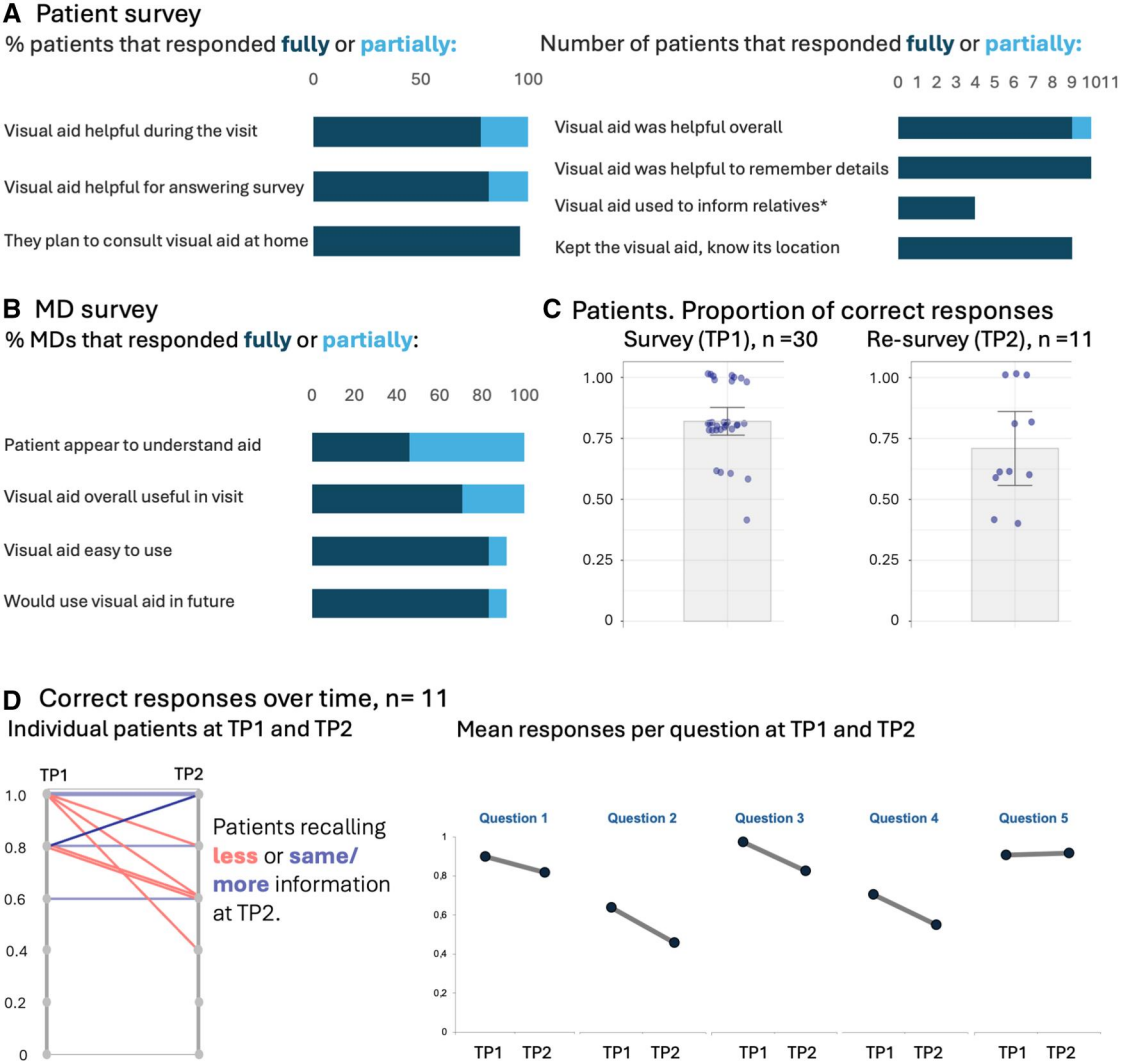
Clinical evaluation of visual treatment timelines. (A) Responses from patients immediately after interview with treatment plan (*n* = 30) and responses from patients at re-survey several weeks after initial interview (*n* = 11). * missing responses: patients had not spoken with any relatives about their treatment. (B) Responses from MDs that used visual treatment timelines for treatment paths in patient interviews, *n* = 24. (C) Proportion of correct responses by patients at outpatient consultation (*n* = 30) and proportion of correct responses from patients at re-survey several weeks after initial interview (recall, *n* = 11). (D) Comparison of individual patients’ responses and question-level proportion of correct responses at survey and re-survey. Questions: see Table S3.

**Table 4.** Summary of results from comparing information delivery formats for cancer treatment paths (Study 2).

Variable	Group P	Group T	Group A	Significant difference between groups, Tukey post-hoc
	Mean (std)	Mean (std)	Mean (std)	Mean difference (95% CI), *P*-value
Proportion correct responses	0.841 (0.117)	0.820 (0.137)	0.676 (0.163)	Picto: Audio 0.14 (0.07, 0.20), <.001 Text: Audio 0.16 (0.10, 0.23), <.001 Text: Picto 0.03 (−0.04. 0.90), .631 *not sig*
Confidence rating	0.78 (0.13)	0.82 (0.11)	0.55 (0.16)	Picto: Audio 0.18 (0.13, 0.22), <.001 Text: Audio 0.20 (.15, 0.24), <.001 Text: Picto 0.02 (−0.03. 0.07), .606 *not sig*
Rating of information quality	0.79 (0.19)	0.76 (0.21)	0.62 (0.23)	Picto: Audio 0.13 (0.06, 0.21), <.001 Text: Audio 0.11 (.04, 0.18), .001 Text: Picto −0.02 (−0.1, 0.05), .868 *not sig*
Total response time (sec)	21.43 (9.22)	21.71 (9.5)	14.23 (5.76)	Picto: Audio: 7,21 (3.56, 10.85), <.001 Text: Audio 7.64 (4.12, 11.61), <.001 Text: Picto 0.43 (−3.95, 4.81), .970 *not sig*
Response time (sec) confidence	4.19 (1.27)	4.82 (2.06)	5.14 (0.90)	No statistical difference (Welch-ANOVA)

Included participants: 160. Groups: A (audio only), P (Audio and Pictogram-based visual aid), T (Audio and Text-based visual aid).

Abbreviation: std = standard deviation.

### Study 3—clinical evaluation of visual treatment timelines

Given that visual timelines improved comprehension of cancer treatment paths, and that visual elements were clear to the majority of participants, including the relevant age group for hematological diseases, we next evaluated the effectiveness of visual aids in the clinic for 3 use-cases: patients treated for multiple myeloma with autologous stem cell transplantation (Figure 1A), patients undergoing allogeneic stem cell transplantation, and patients receiving CAR-T cell therapy (Figure S4). The 30 patients we surveyed (aged 44-72, average 58) were similarly positive, all responded that the aids helped during the interview and for answering questionnaire, and they plan to consult them again (Figure 4A). For the 5 content questions the mean accuracy was 0.82 (sd 0.15) and 5 of the 30 patients answering all questions correctly (Figure 4C, Table 5). All MDs (*n* = 24) fully or partially agreed that patients seem to understand the aids, and that aids were a helpful addition; almost all MDs partially or fully agreed that they were able to use visual aids without preparation and indicate that they would include aids in future communication (Figure 4B).

**Table 5.** Individual patients’ proportion of correct responses over time (Study 3).

	Survey (timepoint 1)—proportion correct responses	Re-survey (timepoint 2) —proportion correct responses	Change
Grouped	Mean (std)	Mean (std)	Delta
All patients	0.82 (0.15)	0.71 (0.23)	
Patient ID	Mean	Mean	Delta
13	1.0	0.6	−0.4
14	0.8	0.8	0.0
15	0.6	0.6	0.0
16	1.0	0.8	−0.2
17	1.0	0.4	−0.6
19	0.8	1.0	0.2
20	1.0	1.0	0.0
21	0.8	0.6	−0.2
28	1.0	1.0	0.0
26	0.4	0.4	0.0
27	0.8	0.6	−0.2

Patients with multiple myeloma return to hospital several weeks after the begin of therapy to undergo stem cell apheresis for the following autologous stem cell transplantation. At this point we were able to re-survey 11 of the initially 30 patients. While we observed a drop in overall proportion of correct responses to 0.71 (sd 0.23, Figure 4C), this is still a high recall rate, and 5 of the 11 patients remembered the same amount as right after the interview (Figure 4D, Table 5). Some questions were easier to answer than others, we therefore also analyzed the question-level success ratio at both survey time points. This revealed that while we saw a drop in the proportion of correct responses for each question, the mean accuracy was still high after several weeks, with the question with lowest score still correctly answered by >50% of patients (Figure 4D). Patients had kept their visual treatment timeline, and still fully or partially agreed that it had helped them understand the procedure. Moreover, they indicated that they had consulted the plan at home, and, if the spoke with relatives about their treatment (4/11), used it to refresh their memory, and even send pictures/photocopies of the plan, to relatives (Figure 4A).

## Discussion

In this work, we investigated to what extent visual treatment timelines communicating the treatment path can effectively supplement health care information. We used visual treatment timelines for 3 hematology treatments as example cases. Survey results reveal that visual treatment timelines significantly enhance comprehension and increase participants’ confidence when responding to content questions on treatment paths, compared to audio alone. Consistent with existing literature,^31^^,^^41^ pictograms and comics often outperformed photo representations and were deemed suitable across various age groups, highlighting their accessibility and versatility in patient communication. Our data also show that some visual representations were not sufficiently guessable, therefore, legibility should be evaluated for each visual representation and, when used, pictograms should be combined with an explanation and a legend.

In our clinical evaluation, MDs and patients positively responded to integrating visual treatment timelines in consultations, and patients remembered treatment details to correctly answer questions immediately as well as several weeks after the interview. Patient consultations take place under time pressure as staff is obliged to provide comprehensive and legally compliant information on various aspects of treatment. The American Cancer Society recommends that patients request decision aids, eg, in the form of written treatment plans or schedules.^37^ Thus, visual aids like our treatment timelines, designed with minimal text and supplemented with pictograms, could effectively complement patient interviews.

Interestingly, we found that the pictogram-based visualization and the text-based treatment timeline were equally helpful for recall among study participants, suggesting that any form of supportive information is better than none.^27^ Research on multimedia learning supports this, indicating that a mixed-format approach is more effective than relying on a single channel.^52^ From an information design perspective, the text-based timeline, despite lacking decorative elements like pictograms or color, qualifies as a visual aid due to its organized layout along an axis.^53^ However, effectiveness of specific visual aids is dependent on the communication goals, which could range from enhancing recall of information to influencing perceptions or encouraging specific health behaviors.^49^ Further research is needed to determine whether a purely text-based description can be just as effective for recall and trust and to understand how readers perceive and differentiate these 2 forms of visual information.

Currently, visual aids that explain general practices, treatment timelines, procedures, or risks, are underutilized in patient consultations.^35^ Although physicians acknowledge the value of visual aids, they are rarely used, mainly because visual aids are not readily available.^35^ This is in contrast to anatomical illustrations that are more common in surgery when patients provide consent for surgery procedures.^54^ Our survey of existing patient information materials for hematological neoplasms revealed that information figures and data visualizations are largely missing in brochures for cancer treatment. This aligns with findings from a previous systematic review.^36^ In aging societies, where the number of elderly cancer patients is still rising, effective healthcare and risk communication present a significant challenge. This challenge could be mitigated by using appropriate visual aids for patients that help the various communication goals in healthcare, from identifying and recalling information, to behavioral adjustments.^49^^,^^55–57^

A limitation of our study is the surveyed demographics in the clinical evaluation, as only a small number of patients were available locally during the recruitment period. The limited number of patients also meant that in this first clinical evaluation we could not randomize patients into 2 arms, a control and an experimental group. However, based on our initial evaluation conducted with the intended audiences,^6^^,^^47^ and with feedback from the patient board, we conclude that the visual cancer treatment timelines do significantly enhance comprehension within our intended audiences group. A logical next step therefore is a multi-centric, controlled clinical trial to pave the way for clinical adoption. In an aging society hematological neoplasms among elderly is still rising^58^ and treatments become more complex with advancements in patient stratification and personalized medicine,^59^^,^^60^ making accessible patient information even more pressing. A trial could also compare several realizations of the visual treatment plan and possibly also test measurable effects on the quality of life.

Our visual treatment timelines likely have broader applications, eg, for other cancer types or other long treatment schedules. Such visual aids may also fill the information need of the elderly patients, experiencing an anxiety-inducing diagnosis ^16^^,^^23^^,^^61–63^ and could generally support vulnerable populations, pediatric patients, relatives and caregivers, nurses.^6^ Given that the online surveys (Study 1-2) were skewed towards a younger population with high health literacy (Study 1) or prior knowledge of cancer treatment (Study 2), our data provides limited insights into vulnerable populations. It is possible that group-based differences, such as variations in response time or confidence, could manifest more subtly. However, the granularity of our study does not allow for a detailed analysis of these potential effects.

The rapid developments of AI-based tools likely will also facilitate generating visual aids from text-prompts. Several resources are already available that offer a wide range of health-related icons/pictograms, which could enhance the visual treatment plans. Examples are Smart Servier, BioIcons, SVGrepo, and Health Icons. Health Icons (healthicons.org/) provides access to over 1300 medical icons for anatomical, disease-related and treatment-related terms and medical devices under a public domain/CC0 license. These icons can be easily integrated into software tools like interactive dashboards or AI-based applications, expanding their utility in healthcare settings. At times, modest measures can have profound effects, as was demonstrated by improved cancer survival when monitoring patients well-being with questionnaires.^64^ Patients expressed gratitude for these visual aids, treasuring them as they navigate their health care journey. The aids provide tangible answers to important questions that were also raised by the patients involved in this study, such as “How long will I be away from home?” and “How often do I come back to the hospital?.”

## Supplementary Material

ocae319_Supplementary_Data

## Data Availability

The data, study materials, and supplementary data are freely available at our Open Science Framework https://doi.org/10.17605/OSF.IO/WKQB4. The protocol for study 1 (Transparency and translucency of visual representations) was preregistered: https://osf.io/cs57n; the protocol for Study 2 (Information delivery formats for cancer treatment timelines) was preregistered: https://osf.io/t2gkq.
