# Supplementary material for: Communicating cancer treatment with pictogram-based timeline visualizations
Source: J Am Med Inform Assoc. 2025 Jan 16;32(3):480–91. doi: 10.1093/jamia/ocae319 (PMC11833489; doi:10.1093/jamia/ocae319)
Supplement: ocae319_Supplementary_Data [file ocae319_supplementary_data.zip › ocae319_Supplementary_Data/SupplementaryTables.pdf]

Supplementary Materials

Supplementary Table 1. Existing patient information.

Comparison of public information for three haematological neoplasm therapies (MM, multiple myeloma; AlloTX, allogenic transplantation for e.g. AML patients; CAR-T, targeted immunotherapy for e.g. lymphoma patients) in USA, UK and Germany (DE). The information includes text only or few visuals with decorative character (figure of cell types, patient). In three cases a process was visualized, each without details.

Organization (Country)	Case	Text	Visual elements (photos, diagrams)	Visual of process
NHS (UK)	MM	Website	-	-
Myeloma UK (UK)	MM	12-page PDF	-	-
Myeloma UK (UK)	MM	58-page PDF	3	-
Cancer research UK	MM	Website	2	-
Cancer research UK	MM	Website	2	1
NCI (USA)	MM	Website	4	-
KID (DE)	MM	Website	1	-
Onkopedia (DE)	MM	Website	4	-
Myelom-net (DE)	MM	60-page PDF	9	-
Myelom.org (DE)	MM	Website	-	-
Lymphome-de (DE)	MM	12-page PDF	2	-
DLH (DE)	MM	50-page PDF	4	-
NHS (UK)	AlloTX	Website	-	-

Cancer research UK	AlloTX	Website	2	1
NCI (USA)	AlloTX	Website		1
KID (DE)	AlloTX	Website	1	-
Onkopedia (DE)	AlloTX	Website	-	-
NCI (USA)	CAR-T	Website	2	-
NHS England (UK)	CAR-T	29-page PDF	-	-
KID (DE)	CAR-T	2-page PDF	1	-
Lymphome-de (DE)	CAR-T	12-page PDF	4	-

Supplementary Table 2.

Summary of **Study 1**, pictogram survey. Median total response times (mimutes:seconds).

		Survey Time	Transparency	Translucency
Time, all		15:06		
Age	<20	17:36		
	20-39	13:37		
	40-59	15:28		
	60+	20:56		
Pictogram, all			01:21	00:45
Age	<20		01:23	00:46
	20-39		01:10	00:42
	40-59		01:29	00:47
	60+		01:54	01:03
Photo, all			01:39	00:50

Age	<20	01:59	00:53
	20-39	01:26	00:46
	40-59	01:46	00:53
	60+	02:41	01:10
Comic, all		01:29	00:49
Age	<20	01:42	00:50
	20-39	01:16	00:45
	40-59	01:37	00:51
	60+	02:09	01:08

Supplementary Table 3.

Question used in Study 2 and 3, and the information queried.

Questions	Patient location	Technical information	Timing of Procedure	Frequency of Procedure
**Study 2** Information delivery formats for cancer treatment timelines				
Q1 - How often is a blood count taken during your first inpatient stay?			X	X
Q2 - When does the bone marrow puncture occur during the first inpatient			X	

stay?				
Q3 - What happens between inpatient stays?	X			
Q4 - When are you in the hospital?	X			
Q5 - How long can the follow-up last?			X	
Q6 - Where are you during the follow-up?	X			
Q7 - How is the chemotherapy administered?		X		
Q8 - How long is the chemotherapy administered?		X	X	
Q9 - When is the chemotherapy administered?		X	X	
Q10 - When does the stem cell transplantation take place?			X	
**Study 3** Clinical evaluation of visual treatment timelines				
Q1 - How long will you be in the clinic for treatment (at least)?	X		X	
Q2 - When will you receive the [autologous blood stem cells/allogeneic blood stem cells/CAR-T cells]?			X	
Q3 - How are the blood stem cells administered?		X		
Q4 - How often do you come for follow-up care to the hospital?			X	X
Q5 - How do you receive the conditioning chemotherapy?		X		

Supplementary Table 4.

Summary of **Study 2 – Comparing information delivery formats for cancer treatment paths,** 160 respondents.

Group	Observation	mean	sd	min	max	range	se
A (Audio)	Proportion correct responses	0.67	0.17	0.3	1	0.7	0.02
	Confidence rating	0.55	0.16	0	1	1	0.02
	Rating of info quality	0.62	0.23	0	1	1	0.03
	Total response time	14.23	5.76	6.59	41.13	34.54	0.74

	Total response time confidence	4.19	0.9	2.8	7.36	4.56	0.12
T (Text)	Proportion correct responses	0.82	0.14	0.5	1	0.5	0.02
	Confidence rating	0.78	0.13	0.5	1	0.5	0.02
	Rating of info quality	0.79	0.19	0.33	1	0.67	0.03
	Total response time	21.43	9.22	9.17	63.37	54.2	1.35
	Total response time confidence	4.82	1.27	3.06	9.08	6.01	0.19
P (Pictogram)	Proportion correct responses	0.84	0.12	0.5	1	0.5	0.02
	Confidence rating	0.82	0.11	0.6	1	0.4	0.01
	Rating of info quality	0.76	0.21	0.33	1	0.67	0.03
	Total response time	21.71	9.5	10.67	55.53	44.86	1.3
	Total response time confidence	5.14	2.06	2.84	13.81	10.97	0.28
