# Supplementary figures and images for: Communicating cancer treatment with pictogram-based timeline visualizations
Source: J Am Med Inform Assoc. 2025 Jan 16;32(3):480–91. doi: 10.1093/jamia/ocae319 (PMC11833489; doi:10.1093/jamia/ocae319)

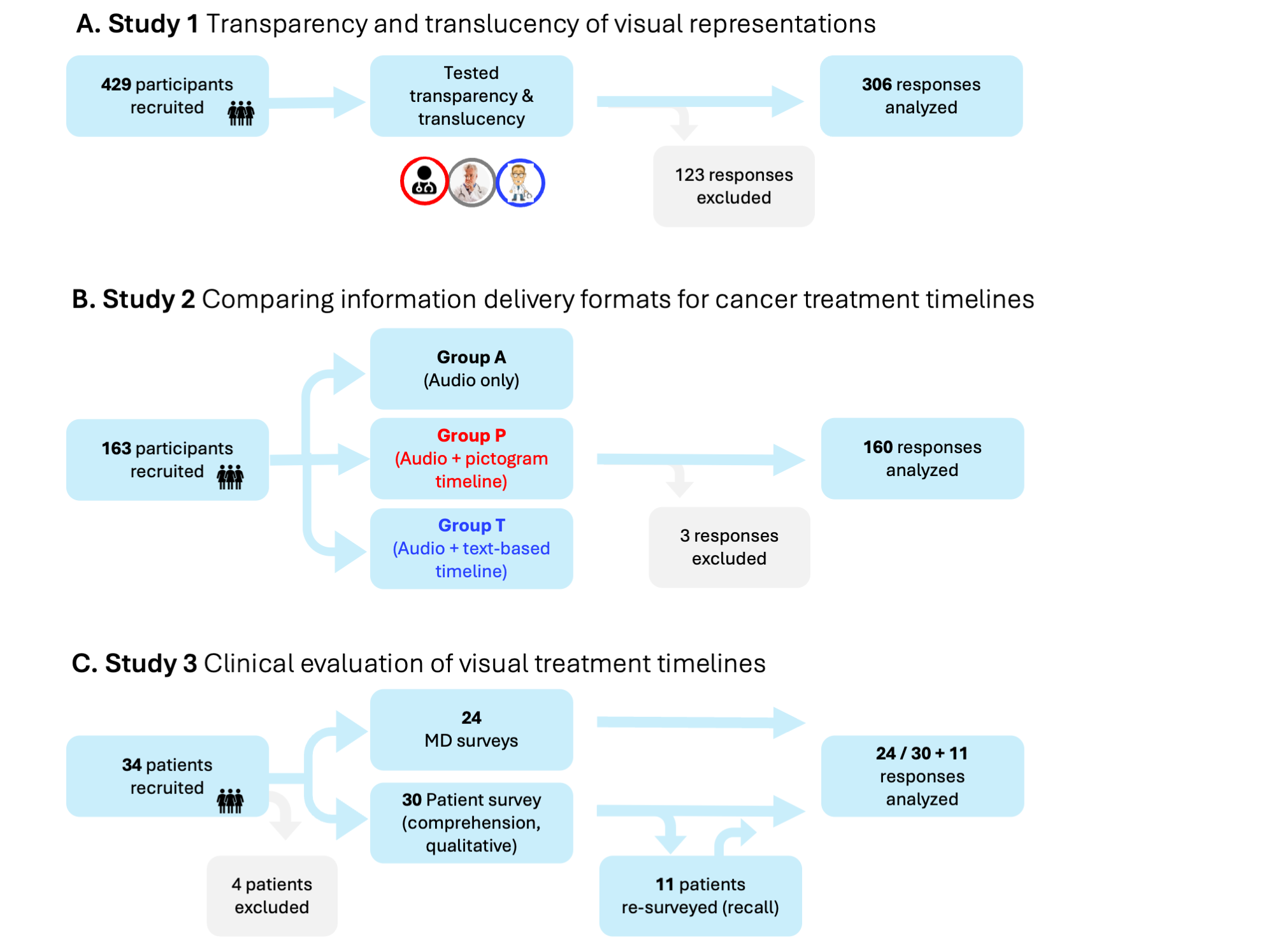

Supplement: ocae319_Supplementary_Data [file ocae319_supplementary_data.zip › ocae319_Supplementary_Data/SupplFig1_Flowchart.png]

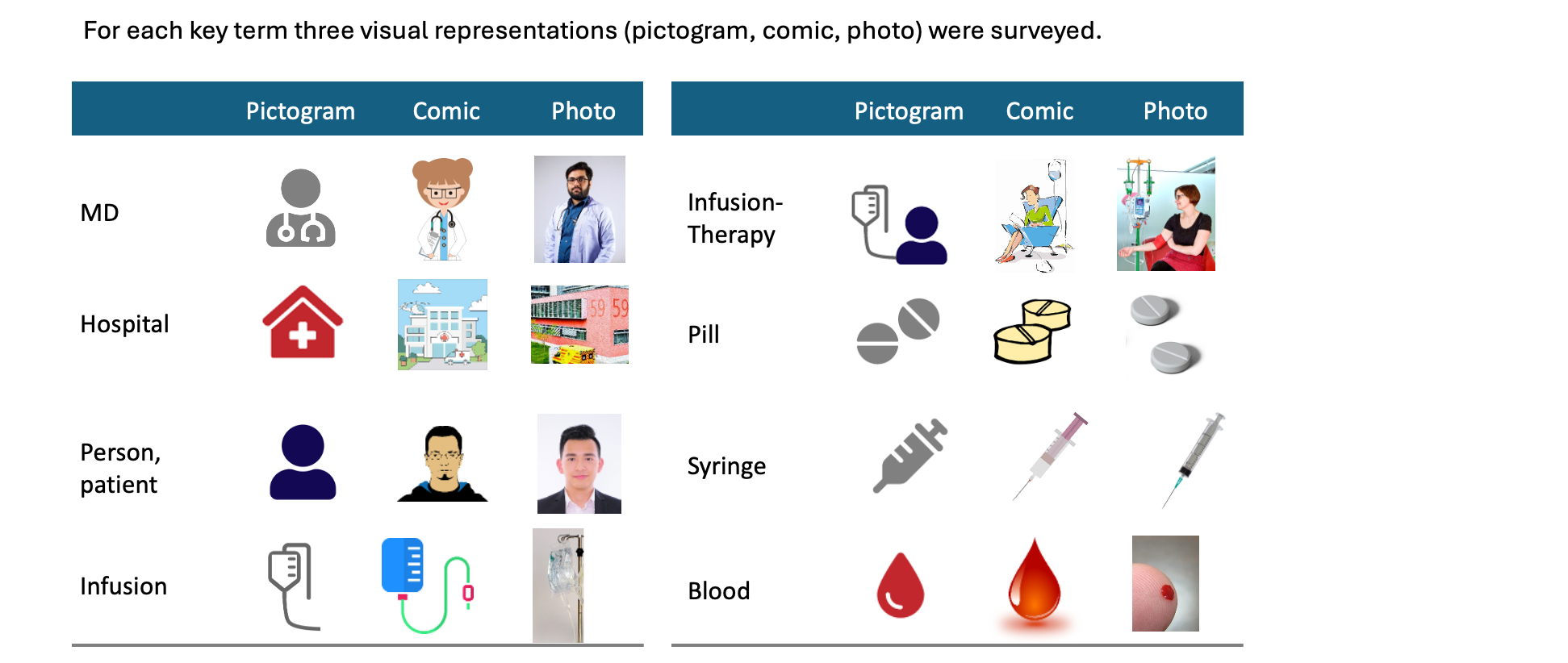

Supplement: ocae319_Supplementary_Data [file ocae319_supplementary_data.zip › ocae319_Supplementary_Data/SupplFig2_VisualRepresentations.png]

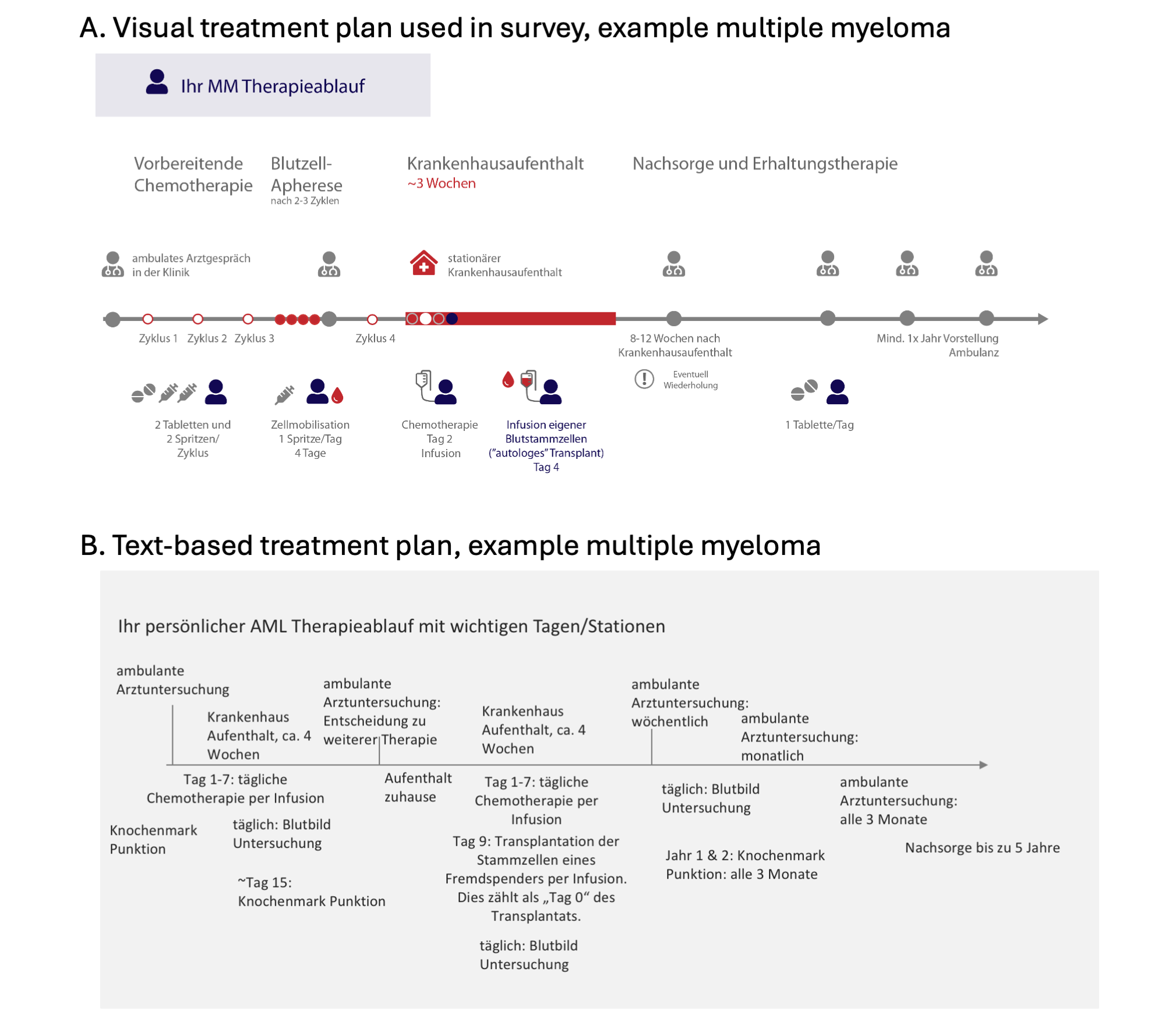

Supplement: ocae319_Supplementary_Data [file ocae319_supplementary_data.zip › ocae319_Supplementary_Data/SupplFig3_TreatmentplansDE.png]

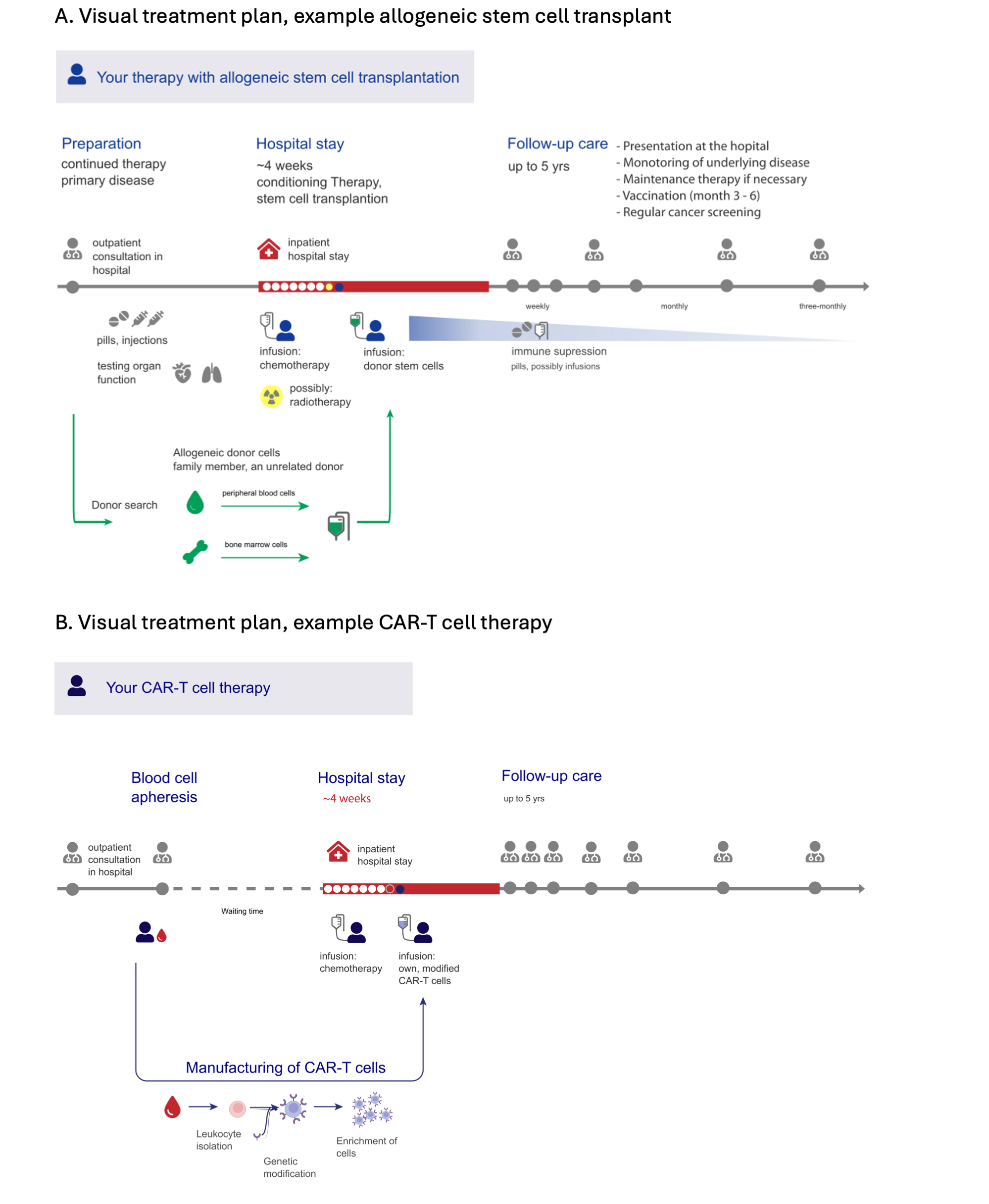

Supplement: ocae319_Supplementary_Data [file ocae319_supplementary_data.zip › ocae319_Supplementary_Data/SupplFig4_Treatmentplans.png]
